# Case of a Ganglion of the Tendons, Opened, and Successfully Treated

**Published:** 1787

**Authors:** John Evans

**Affiliations:** Liverpool


### IX. *Case of a Ganglion of the Tendons, opened, and succesfully treated. By* John Evans, *M. D. of Liverpool.*

MARY Mather, a robust young woman, about twenty-eight years of age, servant in a gentleman's family in this town, applied to me March 5th, 1787, concerning a large ganglion of the tendons, which occupied a considerable part of the back of the left hand, and had been growing gradually larger for the space of four years; but of late had become very painful, being increased to an uncommon size. Upon examination it appeared to be seated on one of the tendons of the extensor communis digitorum, and, passing under the annular ligament of the wrist, extended about three inches above it. Upon pressure being made below the ligament, the contents of the tumour were readily forced underneath it upwards, and by the same means, as easily returned: therefore, before I ventured to make an incision, I got the superior end of the cyst firmly pressed by the hand of an assistant, with a view of forcing whatever it contained into as small a space as possible, and consequently rendering the operation more effectual. Being thus prepared, prepared, I began the incifion below the liga-ment, and continuing it to the inferior edge of the cyft, the whole of that part of it fituated on the back of the hand was laid open; when, to my great aftonifhment, inftead of a glairy fluid, which is common to thefe kind of tumours, I found a number of fubftances, in all about two hundred, of different fizes, refembling, in every refpect, fo many unripe nut kernels. Upon opening fome of them, I found that they contained a pellucid fubftance, fo folid, that it did not eafily yield to preffure between the finger and thumb. The major part of them I have preferved in fpirits, in which they retain their whitenefs and form; but fome of them that were wrapt in paper, and carried by the patient in her pocket to fhow her friends, were, in the fpace of twenty-four hours, fhrivelled to half their original fize, and became as hard and tranfparent as horn.

Inftead of dividing the ligament, or cutting away part of the cyft, according to the practice of fome eminent men *, I deftroyed as much of the internal part of it as feemed neceffary

* See Gooch's Cafes and Remarks in Surgery, Sharpe's Operations, and Warner's Cafes in Surgery.

with lunar cauſtic, and, by the uſe of mild digeſtives and a proper application of bandage, the wound was perfectly healed in three weeks, without any diſagreeable circumſtance intervening.

The above caſe ſeems to confirm the opinion of Mr. Gooch, that the contents of the tumours called ſteatomatous and atheromatous are nothing more than lymph under different degrees of inſpiſſation.

